# Performance and Antiwear Mechanism of 1D and 2D Nanoparticles as Additives in a Polyalphaolefin

**DOI:** 10.3390/nano14131101

**Published:** 2024-06-27

**Authors:** María J. G. Guimarey, Antía Villamayor, Enriqueta R. López, María J. P. Comuñas

**Affiliations:** 1 Laboratory of Thermophysical and Tribological Properties, NaFoMat Group, Department of Applied Physics, Faculty of Physics, University of Santiago de Compostela, 15782 Santiago de Compostela, Spain; enriqueta.lopez@usc.es (E.R.L.); mariajp.comunas@usc.es (M.J.P.C.); 2 Physics of Surfaces and Materials Unit, Tekniker, Basque Research and Technology Alliance (BRTA), C/Iñaki Goenaga 5, 20600 Eibar, Spain; antia.villamayor@tekniker.es

**Keywords:** hexagonal boron nitride, multi-walled carbon nanotubes, polyalphaolefin, friction, wear

## Abstract

This work is focused on the thermophysical and tribological study of eight nanolubricant compositions based on a polyalphaolefin (PAO 20) and two different nanoadditives: multi-walled carbon nanotubes (MWCNTs) and hexagonal boron nitride (h-BN). Regarding the thermophysical properties, density and dynamic viscosity of the base oil and the nanolubricants were measured in the range of 278.15–373.15 K, as well as their viscosity index, with the aim of evaluating the variation of these properties with the addition of the nanoadditives. On the other hand, their lubricant properties, such as contact angle, coefficient of friction, and wear surface, were determined to analyze the influence of the nanoadditives on the tribological performance of the base oil. The results showed that MWCNTs and h-BN nanoadditives improved the wear area by 29% and 37%, respectively, at a 0.05 wt% concentration. The density and dynamic viscosity increased compared with the base oil as the nanoadditive concentration increased. The addition of MWCNTs and h-BN nanoparticles enhanced the tribological properties of PAO 20 base oil.

## 1. Introduction

The increasing use of synthetic lubricants in different industrial applications generates the need to improve these lubricants to reduce the costs associated with friction losses. Commercial lubricants must operate under severe conditions, generating high energy losses due to friction and wear, so it is vital to improve their tribological properties [1]. In particular, the improvement of fuel economy has become very important in the automotive sector. One of the main ways to address this issue is to reduce friction losses in the engine, for which the properties of engine lubricants have been improved by using different additives [2,3,4]. Synthetic engine lubricants containing polyalphaolefins (PAO) as base oils have been widely used. The main reason for the use of PAOs is their high-performance lubrication owing to their myriad advantageous attributes, including mechanical and thermal stability that protects equipment subjected to rigorous performance demands, elevated viscosity index that facilitates their use in both extreme hot and cold conditions, minimal volatility, and low pour point [5]. In addition, improved fuel economy and energy efficiency, as well as reduced overall costs, are the main reasons for the use of PAOs as base oil [6].

Employing nanomaterials as additives in base oils stands out as a highly efficient strategy for managing friction and wear, holding immense importance for energy preservation, emission mitigation, and environmental safeguarding. Nanomaterials are attractive as lubricant additives due to their tiny size, facilitating easy infiltration into friction contact zones and the subsequent formation of a protective tribofilm [7,8]. This tribofilm serves to deter wear on the surfaces of frictional pairs. Moreover, nanomaterials exhibit high surface activity, enhancing the stability of the protective tribofilm formation through physical and/or chemical adsorption effects. According to Zhao et al. [9], there are three basic types of nanolubricant additives: nanometal-based additives, nanocarbon-based additives, and nanocomposite-based additives. Among the nanocarbon-based additives, one-dimensional (1D) multi-walled carbon nanotubes (MWCNTs), owing to their shape, high length-to-diameter ratio, great flexibility, and high electrical and thermal conductivities, are promising for their use as solid lubricants [10,11,12]. Zhang et al. [13] found that MWCNTs readily opened and peeled into graphene-like lamellar layers under high loads, resulting in favorable tribological properties through an interlayer-sliding mechanism.

Another nanoadditive that has attracted a lot of interest is hexagonal boron nitride (h-BN) due to its encouraging lubricating properties, thermal stability, and chemical inertness [14]. h-BN features a two-dimensional (2D) layered crystalline structure akin to that of graphite. Within each layer, atoms are bonded together by strong covalent bonds, whereas the layers themselves are held together predominantly by weak van der Waals forces [15]. h-BN offers a potential alternative to graphite or MoS_2_ in broader applications as an additive lubricant [16] and was classified as a clear additive. Çelik et al. [17] have investigated the effect of h-BN nanoparticles as a lubricant additive in engine oil (SAE10 W), studying the friction and wear behavior of AISI 4140 steel. These authors have concluded that the addition of h-BN increased the friction coefficient by 14.4% while decreasing the wear rate by 65%.

The tribological properties of lubricant formulations that use polyalphaolefins (PAOs) as base oils and nanometal-based additives have been investigated in previous works [18,19,20,21,22,23,24,25,26,27,28]. Jeng et al. [29] have published experimental research on the tribological properties of mineral oils containing carbon nanocapsule (CNC) additives. These authors observe that the addition of CNCs to the mineral oil yields an effective reduction in the friction coefficient at the contact interface. Jeng et al. [30] have also reported the tribological properties under high contact loads of mineral oil lubricants containing carbon-Fe nanocapsules (CFNCs), obtaining for a load of 650 N and a CFNC concentration of 0.07 wt% an improvement of the microbearing lubrication. Bukvić et al. [31] published a review article on the tribological properties of nanocomposite-based lubricants, focusing on carbon nanotubes. Concerning the use of multi-walled carbon nanotubes or hexagonal boron nitride as additives to polyalphaolefins, Kumar and Harsha [32] have determined the friction, antiwear, and extreme pressure properties of COOH-functionalized multi-walled carbon nanotubes dispersed in four different polyalphaolefins (PAO 4, PAO 6, PAO 40, and PAO 100) at various concentrations (0.025, 0.05, 0.075, 0.10, and 0.15 wt%). These authors have concluded that by adding the optimum concentration of MWCNTs in all PAOs except PAO 4, the surface topography of worn surfaces improved significantly. In other work, Kumar et al. [33] have also studied the tribological performances of COOH-functionalized multi-walled carbon nanotubes (MWCNTs) dispersed in two different polyalphaolefins (PAO 4 and PAO 6) at a dose of (0.025–0.15 wt%).

Liñeira del Río et al. [34] determined the friction coefficient and the wear for PAO 40 additive with 0.25, 0.50, 0.75, and 1.00 wt% of h-BN and 0.05, 0.10, 0.25, and 0.50 wt% of GnP. These authors observed that for the optimal concentration (0.75 wt% of h-BN and 0.50 wt% of GnP), the friction coefficient reduces by around 20%. Xia et al. [35] reported the tribological properties at different speeds and under various loads of nanolubricant samples containing hexagonal boron nitride (h-BN), carbon nanofibers (CNFs), and carbon nanotubes (CNTs) dispersed in a PAO. These authors used higher nanoadditive concentrations, finding the lowest and most stable average friction coefficient for the sample containing 6-wt% CNF and 6-wt% h-BN. Jiang et al. [36] studied the physicochemical properties of h-BN nanoparticles dispersed in PAO 6. They analyzed the colloidal stability of the nanoparticle suspension, the influence of shear rates on shear stress and viscosity, and the effects of temperature and nanoadditive concentration on viscosity. These authors have also compared the tribological behavior of steel/steel surfaces lubricated with PAO 6 oil containing h-BN and other hybrid nanoparticles (MoS_2_/h-BN), using oleic acid as a surfactant in the formulation. Nasser et al. [37,38,39] determined the tribological properties of PAO 32 additive with h-BN or GnP at a concentration of 0.025, 0.05, or 0.1 wt%. These authors have also studied hybrid nanolubricants used in the formulation, h-BN, graphene nanoplatelets, and ionic liquids. Recently, Ziyamukhamedova et al. [40] analyzed the impact of h-BN nanoparticles on the rheological and antiwear characteristics of several base oils, among them PAO 4. Finally, we should remark that we can also find in the literature other publications [41] focusing on the prediction of the thermophysical properties of polyalphaolefin + hexagonal boron nitride using machine learning models.

An analysis of the previously cited [17,18,19,20,21,22,23,29,30,31,32,33,34] articles on the tribological properties of PAOs + h-BN or MWCNTs initially reveals some contradictions. For example, the results obtained by Çelik et al. [17] and Liñeira del Río et al. [34] indicate, in the first case [17], an increase in the friction coefficient due to the presence of h-BN, but in the second case [26], just the opposite, for the optimal nanoparticle concentration. However, we must point out that it is difficult to establish comparisons between different lubricant formulations. Thus, Çelik et al. use as base oil an engine oil, in which surely other additive packages are present; on the contrary, Liñeira del Río et al. use as base oil only PAO40 without other commercial additives. The additive package, which can include other compounds with the aim of improving other lubricant properties (such as pour point, thermal stability, viscosity grade, or viscosity index, among others), can also affect the tribological properties of the final lubricant. In addition to the synergies with other additives used in the formulation of the lubricant, the effect of the functionalization and synthesis of the nanoparticles, the concentration used in the formulation, the temporal stability, of the nanoparticle agglomeration on the tribological mechanisms, together with the impact of nanoparticles on the environment and the costs of the use of some nanoparticles, need more comprehensive understanding, addressing the need for more studies in this field.

The present work reports frictional results and discusses the possible wear mechanism to improve the tribological behavior of a polyalphaolefin, PAO 20, containing hexagonal boron nitride nanoparticles, h-BN, or multi-walled carbon nanotubes, MWCNTs. As far as we know, this is the first article studying the tribological performance of PAO 20 with MWCNTs and h-BN. To check the influence of the nanoparticles on the thermophysical properties of the nanolubricants, a thermophysical characterization was carried out, measuring their density, viscosity, and viscosity index (VI). The effect of the nanoadditives on the wettability of the base oil has been evaluated by determining the contact angle.

## 2. Materials and Methods

### 2.1. Materials

#### 2.1.1. Base Oil

**Figure 1:**
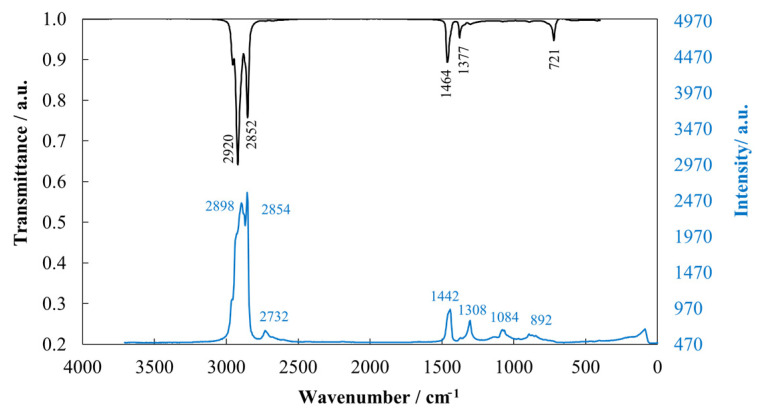
FTIR spectrum (black) and Raman spectrum (blue) of polyalphaolefin (PAO 20) base oil.

The polyalphaolefin, PAO 20, was kindly supplied by Repsol S.A. (Madrid, Spain). This base oil is a mixture of PAO 40 (65 wt%) and PAO 6 (35 wt%), both of which are chemically synthesized through the hydrogenation of polymerized 1-decene. MALDI-TOF mass spectroscopy of the PAO 20 was previously reported by Coelho de Sousa Marques et al. [42], where the results show that the average molecular mass, M_w_, and polydispersity index, M_w_/M_n_, are 627.34 g mol^−1^ and 1.031, respectively. This base oil was further characterized by different techniques, such as infrared spectroscopy (IR) with an FTIR Varian 610-IR spectrometer (Varian, Palo Alto, CA, USA) and Raman microscopy using a WITec alpha300R+ confocal microscope (WITEC, Ulm, Germany) with a 50x objective lens. The laser power was kept at 12 mW with a grating of 600 line/mm. The integration time for each measurement was set to 0.3 s, with 150 accumulations. Figure 1 shows the FTIR spectrum of PAO 20, which reveals the typical bands for polyalphaolefins obtained previously by Guimarey et al. [43] for PAO 6, Nasser et al. [37] for PAO 32, and Liñeira del Río et al. [44] for PAO 40. The Raman spectrum of PAO 20 can be observed in Figure 1. An intense Raman band is detected around 2800–3000 cm^−1^. The broad features in this region correspond to the C-H stretching vibrations of aliphatic hydrocarbons. Specifically, we observe peaks at 2854 and 2898 cm^−1^ assignable to symmetric C-H stretching of methylene and methyl groups, respectively [45]; other peaks at 1308 cm^−1^ and 1442 cm^−1^ correspond to δ(CH_2_) and δ(CH_3_) vibrations, respectively [46]. Additional peaks at 892 and 1084 cm^−1^ can be attributed to the stretching of C-C and C-H and ν(CC) aliphatic chain vibrations, respectively [46,47].

#### 2.1.2. Nanoadditives

Hexagonal boron nitride (h-BN) nanoparticles provided by Iolitec (GmbH, Heilbronn, Germany) present a mole fraction purity of 0.99 (lot MNC018001) a nominal diameter of 70 nm, and a bulk density of 2.29 g·cm^−3^. The h-BN nanoparticles used as additives in the current work were previously characterized by Liñeira del Río et al. [48] and Guimarey et al. [43]. Multi-walled carbon nanotubes (MWCNTs) supplied by NANOCYL^®^ (NC3100™ series, Belgium) were the other nanoadditive employed in this work. MWCNTs were manufactured via the Catalytic Chemical Vapor Deposition (CCVD) process and then purified up to 95 wt%. These nanoadditives were characterized in the present work by both Scanning and Transmission Electron Microscopy (SEM and STEM), Fourier-transform infrared (FTIR) spectroscopy, and Raman microscopy.

**Figure 2:**
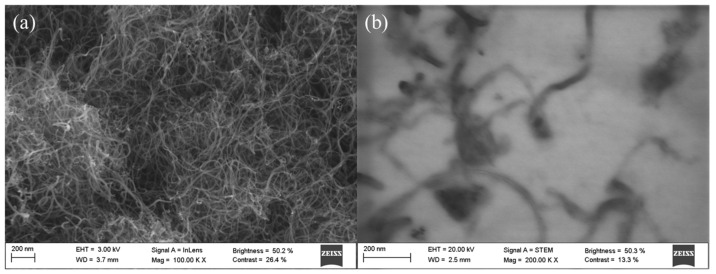
SEM (**a**) and STEM (**b**) images of the multi-walled carbon nanotubes (MWCNTs).

SEM and TEM micrographs were taken with a Zeiss Ultraplus Field Emission Scanning Electron Microscope (FESEM, Carl Zeiss Microscopy GmbH, Jena, Germany). For TEM images, a transmitted electron detector (JEOL JEM-2010, Tokyo, Japan) was used with a voltage of 20–30 kV. For this technique, the carbon nanotubes were dispersed in 1-butanol. The SEM and STEM micrographs shown in Figure 2 confirm the typical tubular morphology of MWCNTs. A large number of long and fine MWCNTs are observed, with the average length and diameter of the tubes being 1.5 μm and 9.5 nm, respectively.

**Figure 3:**
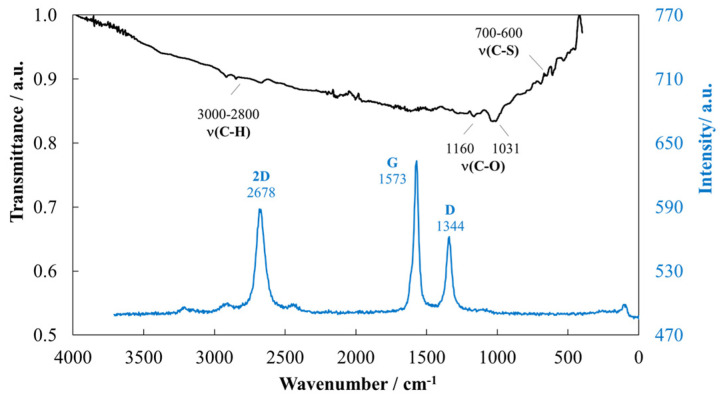
FTIR spectrum (black) and Raman spectrum (blue) of the multi-walled carbon nanotubes (MWCNTs).

The FTIR spectrum of MWCNTs (Figure 3) shows two distinct modes at 1160 and 1031 cm^−1^ assigned to ν(C–O) that is a characteristic vibration mode for CNT–COOH, ester, ether, phenol, or carboxyl groups [49]. In addition, in the range of 3000 to 2800 cm^−1^ and 700 to 600 cm^−1^, some bands are detected corresponding to ν(C–H) vibration characteristic of CNT, CH_2_, and CH_3_ alkyl chains and corresponding to ν(C–S) vibration due to CNT and sulfonic acid groups from using H_2_SO_4_ in the oxidation and purification processes, respectively [49]. The Raman spectrum for the MWCNTs was also performed using a WITec alpha300R+ confocal Raman microscope irradiated at 532 nm. As shown in Figure 3, the Raman spectrum reveals the features bands of carbon-based compounds, including a sharp G-band at 1573 cm^−1^ characteristic of graphitic sp^2^ materials [50], though its slight broadening suggests the presence of multiple layers and some degree of interlayer interaction, typical of few-layer graphene or graphite. The D-band at approximately 1344 cm⁻¹ indicates the presence of defects, which are more prevalent in graphite-like structures due to the higher occurrence of grain boundaries and structural imperfections. The 2D (G′) band is present at 2678 cm^−1^ but broader and more complex than what is expected for monolayer graphene, pointing toward a multilayer structure where interlayer coupling affects the phonon resonance. D and G peaks present an intensity ratio (I_D_/I_G_) of 0.89, less than 1, consistent with graphite and few-layer graphene, suggesting a material with multiple layers rather than single-layer graphene. This ratio can be higher for a MWCNT sample with more defects or less purity [51]. Raman analyses were conducted to study the formation of chemical bonds between nanoadditives and the base oil. Figure S1 shows the Raman spectra for the nanolubricant compositions of the highest nanoadditive concentration for both studied nanoadditives. Raman spectra of the nanolubricants are similar to that of the base oil. No new spectral peaks or shifts are found for the nanolubricant compositions in comparison with the PAO 20 base oil, which indicates that no chemical bonds were formed. This suggests a physical dispersion where the nanoadditives are present but not altering the chemical structure of the base oil.

### 2.2. Design and Stability of the Nanolubricants

**Figure 4:**
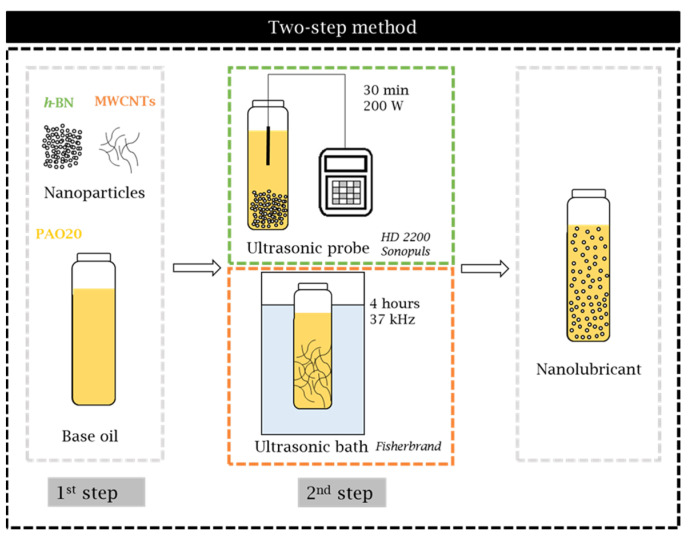
Diagram of the two-step method used to formulate the nanolubricants.

The process for preparing the nanolubricants is schematically represented in Figure 4. The dispersion device employed was different depending on the type of nanoadditive; thus, for h-BN, an ultrasonic probe (HD 2200 Sonopuls, Bandelin, Germany) was used, while for MWCNTs, an ultrasonic bath was used (Ultrasonic bath FB11203 Fisherbrand from Fisher Scientific, Hampton, VA, USA). Further description of the two-step method is available in previous works [43]. Four different concentrations were prepared with each nanoadditive: 0.05, 0.10, 0.25, and 0.50 wt%.

The nanolubricants stability was evaluated by the temporal evolution of the refractive index. This method records the refractive index, *n*, of the nanolubricant as a function of time. For this, a Mettler Toledo refractometer RA-510M (Barcelona, Spain) was used with a glass lens where the sample was deposited. If the nanolubricant is stable, *n* remains constant; otherwise, *n* increases because the nanoadditive is covering the lens.

### 2.3. Thermophysical Characterization

The density and dynamic viscosity of the nanolubricants were measured from 278.15 to 373.15 K and at atmospheric pressure using a rotational Stabinger viscometer SVM 3000 from Anton Paar (Graz, Austria). The automated viscometer is based on a modified Couette principle and includes a vibrating tube densimeter. In addition, this equipment allows the determination of the viscosity index (VI). This device has been previously described in detail [52]. The expanded uncertainties (k = 2) are 0.0005 g·cm^−3^ and 1% for density and dynamic viscosity, respectively. For the temperature in the range of 288.15 to 378.15 K, the expanded uncertainty is 0.02 K, and outside this range, 0.05 K.

### 2.4. Wettability

The contact angle of PAO 20 and the eight nanolubricant compositions was determined using a contact angle analyzer (Phoenix MT(A), SEO, Suwon-si, Republic of Korea) at 293.15 K. Sessile drop method was employed to determine the contact angle values, and method validation was performed using a certified drop calibration reference tool. Prior to conducting the static contact angle measurements, the surfaces were rinsed with ethanol and dried in a stream of hot air. AISI 52100 steel discs were selected as the surface to determine the wetting behavior of the base oil and nanolubricants, as this is the surface material used for tribological tests. In order to determine the average steady-state contact angle, a 30-s waiting period was observed to allow the droplet to stabilize, and at least three measurements were taken for each sample. The static contact angle was determined with an expanded uncertainty of 1 degree and a confidence level of 95%.

### 2.5. Tribological Assays

Rotational friction tests were conducted using a CSM Standard tribometer in a ball-on-disc configuration (CSM Instruments, Peseux, Switzerland), evaluating both the PAO 20 base oil and the formulated nanolubricants. The experimental conditions included room temperature (~298 K), a load of 20 N (equivalent to 2.0 GPa maximum contact pressure), a disc radius of 3 mm, a sliding distance of 340 m, and a speed of 0.10 m s^−1^. Detailed specifications of the tribological specimens (balls and discs) can be found in Table 1. Prior to the friction tests, both specimens were cleaned in an ultrasonic hexane bath and dried with air. Approximately 0.2 mL of lubricant was used for each test to ensure consistency, with a minimum of three replicates performed for each sample to ensure repeatability.

After conducting the friction tests, the wear on the discs was assessed using an Optical 3D Profiler Sensofar S Neox (Terrassa, Spain) in confocal mode (10× magnification). The evaluation involved quantifying wear based on the cross-sectional area. Specifically, the wear area was measured at three distinct zones of the worn surfaces to ensure representative values. Additionally, using the same equipment, the roughness (Ra) of the worn disc surfaces was evaluated to investigate the antiwear mechanisms of the nanolubricants. This analysis followed the ISO 4287 standard, employing a Gaussian filter with a wavelength cut-off of 0.08 mm. In addition, a WITec alpha300R+ confocal Raman microscope was utilized to gather compositional data on the tribofilm within the abraded track, while scanning electron microscope (SEM) analyses were performed using a Zeiss Ultraplus Field Emission (FESEM) instrument to examine the morphology of the worn surfaces. Raman mapping was recorded using a 532 nm, 8 mW laser with an integration time of 0.3 s. The equipment had a grating of 600 g/mm. A Zeiss EC Epiplan-Neofluar 100×/0.9 NA objective was used. The equipment had a 1024 × 127 pixel CCD detector cooled to −60 °C. The spectra correspond to those recorded point to point in a selected area of 75 × 75 microns where records were made in 150 lines and each line 150 points, with a resolution of 0.5 microns in both the x and y axis. A total of 22,500 spectra were obtained, and after eliminating the cosmic rays and correcting the baseline, a Raman image by component distribution was obtained using the WITec Project Five 5.3 software.

## 3. Results

### 3.1. Stability Assessment of Nanolubricants

**Figure 5:**
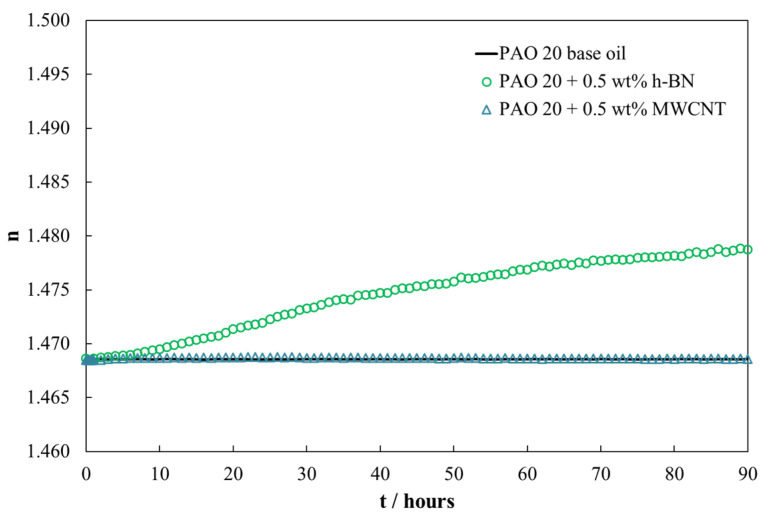
Temporal evolution of refractive index, n, for PAO 20 + 0.5 wt% MWCNT and PAO 20 +0.5 wt% h-BN.

Figure 5 shows the temporal evolution of the refractive index (*n*). It can be clearly observed that the nanolubricant with MWCNT nanoadditives reveals remarkable stability during 90 h of its preparation. On the contrary, the nanolubricant with h-BN shows poorer stability, with a variation of *n* around 0.7% during the first 90 h. The h-BN nanolubricant can be considered stable during the first 12 h, with a variation of *n* of 0.08%. Previous works [53,54] have reported that this variation in refractive index is acceptable for thermophysical and tribological characterization tests.

### 3.2. Thermophysical Properties

**Figure 6:**
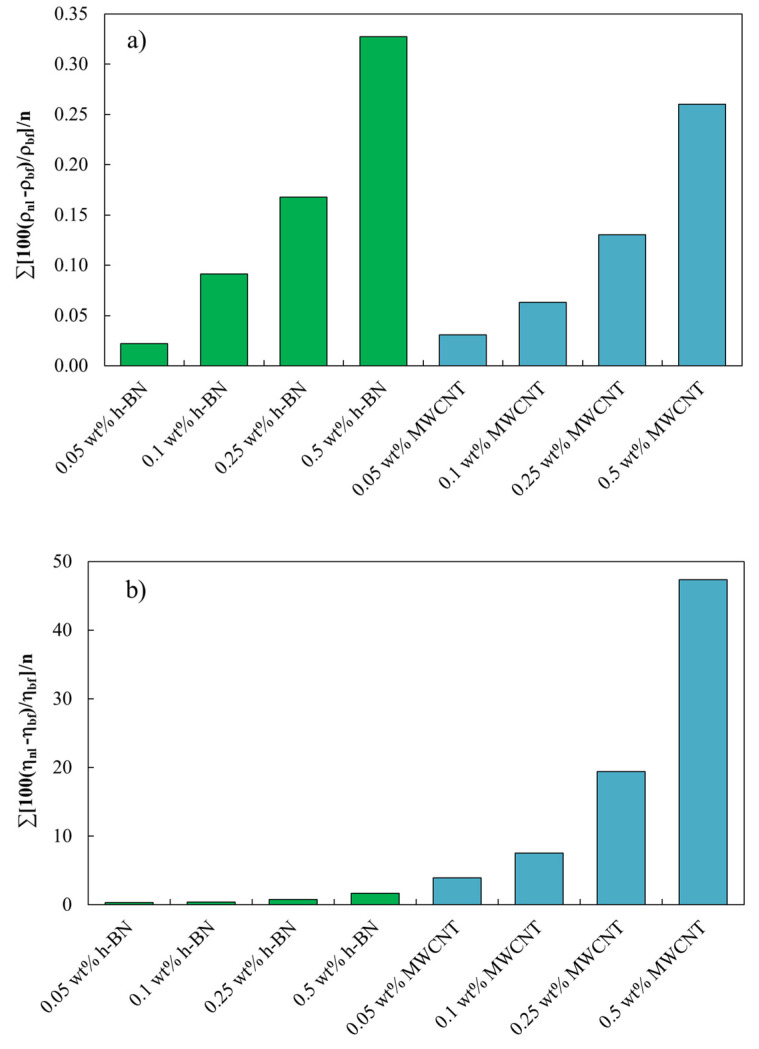
Increments for the eight nanolubricant compositions with respect to PAO 20 neat base oil overall the temperature interval (**a**) density and (**b**) dynamic viscosity.

Density values for PAO 20 base oil and all nanolubricants measured from 278.15 to 373.15 K are presented in Table S1 (Supplementary Materials). Figure 6a shows the density variations obtained for the different nanolubricants as a function of concentration in relation to the unadditivated lubricant. The density increases as the nanoparticle concentration increases. The density increase is higher for h-BN-based nanolubricants than for MWCNT-based nanolubricants for the same nanoadditive loading. For instance, an increase of 0.33% was observed for h-BN/PAO 20 nanolubricants at 0.5 wt%, while for the same MWCNT/PAO 20 concentration, only 0.26% was reached.

Dynamic viscosity results are gathered in Table S2 for the PAO 20 base oil and h-BN- and MWCNTs-based nanolubricants. The relative viscosity variations for the h-BN- and MWCNTs-based nanolubricants compared to PAO 20 neat base oil are plotted in Figure 6b. It can be clearly observed that there is an increase in the dynamic viscosity as the nanoparticle concentration increases. These increments are higher for the MWCNT-based nanolubricants at the same mass concentrations. Thus, for 0.05 wt%, 0.1 wt%, 0.25 wt%, and 0.5 wt% of h-BN, the average absolute deviations (AADs) of dynamic viscosity compared to that of PAO 20 base oil were up to 0.3, 0.4, 0.8, and 1.7%, respectively, while for the same concentrations of MWCNT AADs of 4, 8, 19, and 47%, respectively, were obtained. One possible explanation for this phenomenon could be related to the interaction between the polyalphaolefin (PAO 20) molecules and the different nanoparticles, MWCNTs, and h-BN. So, when MWCNTs are added to PAO 20, their high aspect ratio and surface chemistry may promote strong interactions with the PAO molecules, while h-BN, known for its excellent lubricating properties due to its low shear strength and lamellar structure, may not form as strong interactions with the PAO molecules. As a result, the addition of h-BN may not lead to such a significant increase in effective molecular size or entanglement of PAO molecules compared to MWCNTs, resulting in a lower increase in viscosity. Moreover, the dispersion characteristics of the nanoparticles in the PAO matrix could also play a role. MWCNTs tend to form more stable and uniform dispersions compared to h-BN as we can confirm in Figure 5, so they may have a greater impact on viscosity due to their more effective interaction with the PAO molecules throughout the bulk of the mixture.

The equation based on Vogel-Fulcher-Tammann (VFT) was used to correlate the experimental viscosity data [54], as follows:

ln η(T) = ln A + (B/(T − C))(1)

The parameter values (A, B, and C) are reported in Table S3. This equation reproduces the experimental viscosity values for h-BN-based nanolubricants and MWCNT-based nanolubricants with AADs% lower or equal to 0.40%.

**Figure 7:**
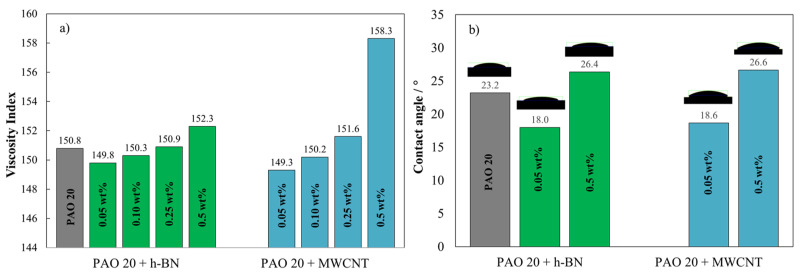
(**a**) Viscosity index (VI) and (**b**) average contact angle for the PAO 20 base oil and for the eight nanolubricant compositions.

Figure 7a shows the viscosity index (VI) for the PAO 20 base oil and for the eight nanolubricant compositions of h-BN and MWCNTs. It can be revealed that both h-BN and MWCNT nanoadditives positively impact the VI of the lubricant when added to the base oil at high concentrations (0.25 wt% and 0.5 wt%). However, MWCNTs, with their superior mechanical properties and lubricating efficiency, lead to a more substantial increment in VI compared to h-BN nanoparticles, reaching an increase of up to 5% for the 0.5 wt% concentration of MWCNTs. Similar results were previously reported by Pourpasha et al. [55] for MWCNT nanolubricants based on turbine oil obtaining VI increased by 2.43% at 0.3 wt% MWCNT compared to the base oil.

### 3.3. Wetting Behavior

Effective surface wetting during contact is pivotal in mitigating friction and wear [56,57]. The wetting behavior of the designed nanolubricants was evaluated by means of contact angle measurement. The average contact angle (θ) value obtained for PAO 20 at room temperature is 23.2°. This value agrees with the data reported by Coelho de Sousa Marques et al. [42] at 293.15 K, θ = 22.5°. Figure 7b shows the average contact angle for the PAO 20 base oil and for the lowest and highest nanolubricant concentrations for both nanoadditives (h-BN and MWCNT). As can be seen, the effect of the nanoadditives on the contact angle of the base oil is directly dependent on the concentration. At low concentrations of h-BN or MWCNT (0.05 wt%), the average contact angle of PAO 20 decreases; however, for high concentrations (0.5 wt%), its value increases. No remarkable difference is observed depending on the nature of the nanoadditive (h-BN or MWCNT) the reductions and increases are very similar for both nanoadditives at the same concentration. For instance, for the 0.05 wt% concentration, the average contact angle is reduced by 22% and 20%, while for the 0.5 wt% concentration, it increases by 14% and 15% for the h-BN and MWCNT modified nanolubricants, respectively. This implies that the wettability of PAO 20 base oil improves at low concentrations of nanoadditives.

### 3.4. Tribological Performance

The lubrication regime for elliptical contacts, at the beginning of the tribological test was determined using Equations (2) and (3), both suggested by Hamrock and Dowson [58].

(2) $$h_{min}=3.63{\left(\frac{U\;\eta }{\;E\;R}\right)}^{0.68}{\left(\alpha \;E\right)}^{0.49}{\left(\frac{F}{E\;R^{2}}\right)}^{-0.073}\left(1-e^{-0.68k}\right)\;R$$

(3) $$\lambda =\frac{h_{min}}{\sigma_{rms}}=\frac{h_{min}}{\sum R_{q}}=\frac{h_{min}}{\sqrt{R_{q1}^{2}+R_{q2}^{2}}}$$

where *h_min_* is the minimum oil film thickness, *R* is radius of curvature of the ball, *U* is the entraining surface velocity, *η* is the dynamic viscosity of the base oil at test temperature (298.15 K) and atmospheric pressure, *α* is the pressure-viscosity coefficient, F is the contact load (20 N), E is Young’s modulus, *σ_rms_* is the root mean square roughness of the ball and disk surfaces, R_q1_ is the surface roughness of the ball and *R_q_*_2_ is the surface roughness of the disk and λ is the ratio between *h_min_* and *σ_rms_*, which may describe the regime of lubrication. The value of α was estimated by using the following correlation proposed by Gold et al. [59], which is based on a database of 28 lubricants, including mineral, synthetic, and vegetable oils:

(4) $$\alpha =s\nu^{t}$$

where *v* is the kinematic viscosity at atmospheric pressure and the operating temperature, and *s* and *t* are the two parameters reported by the authors for polyalphaolefin (PAO) (*s* = 7.382 and *t* = 0.1335). At the given operating conditions, the *λ* value obtained from Equation (3) was 2.67, indicating a mixed lubrication (ML) regime [60].

**Figure 8:**
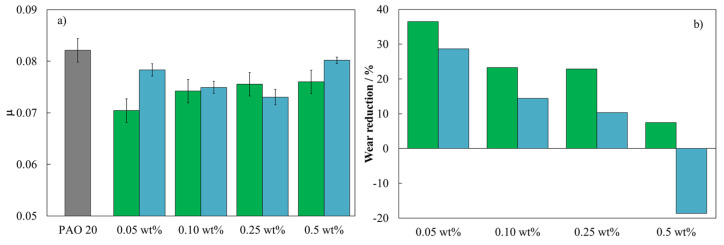
(**a**) Average coefficient of friction and (**b**) wear area reduction for the eight nanolubricant compositions: h-BN (green bars) and MWCNT (blue bars) compared to PAO 20 neat base oil.

The coefficient of friction results obtained for PAO 20 base oil and h-BN and MWCNT-based nanolubricants are plotted in Figure 8a. The coefficient of friction of PAO 20 base oil was reduced with the addition of both nanoadditives at all concentrations. Reductions of 14, 10, 8, and 7% were obtained for the 0.05 wt%, 0.10 wt%, 0.25 wt%, and 0.5 wt% h-BN concentrations, and 5, 9, 11, and 2% for the 0.05 wt%, 0.10 wt%, 0.25 wt%, and 0.5 wt% MWCNT concentrations, respectively. The highest reductions were found for concentrations of 0.05 wt% of h-BN and 0.25 wt% of MWCNT, reaching coefficients of friction of 0.070 and 0.073, respectively. Therefore, the optimum antifriction concentration was found to be the lowest concentration of h-BN. This remarkable anti-friction behavior may be due to the following factors: tribofilm formation, rolling effect, or transformation of the microstructure [13,48]. Liñeira et al. [48] have revealed the best anti-friction performance using h-BN nanolubricant at 0.75 wt% as an additive of trimethylolpropane trioleate base oil, achieving 25% reductions in the coefficient of friction. On the other hand, the anti-friction performance of MWCNTs can be explained by the appropriate diameter (5–20 nm) and length (0.5–20 μm) that facilitate the entry of MWCNTs into the free space of the friction pairs, playing the role of bearing between the friction pairs and filling the cracks to repair the surface damage of the friction pairs [61]. Joly-Pottuz and Ohmae [62] investigated the impact of multi-walled carbon nanotubes (MWCNTs) on the tribological properties of polyalphaolefin (PAO) base oil. Their findings revealed that incorporating 0.1-wt% MWCNTs reduced the friction coefficient to 0.15 at a contact pressure of 0.83 GPa. Moreover, with increasing pressure from 1.42 to 1.72 GPa, the friction coefficient could be further decreased to 0.06.

After sliding friction tests, the cross-sectional area on the steel discs was evaluated. Figure 8b shows the reduction in wear area produced on the steel discs for the h-BN and MWCNTs-based nanolubricants compared to the PAO 20 base oil. A clear reduction of the wear area is observed for most of the concentrations used, except for the highest concentration (0.5 wt%) of MWCNTs. Wear area reductions were 37, 23, 23, and 7% for the 0.05 wt%, 0.10 wt%, 0.25 wt%, and 0.5 wt% concentrations of h-BN, respectively, while for MWCNT nanolubricants, the wear area reductions obtained were 29, 14, and 10% for the concentrations of 0.05 wt%, 0.10 wt%, and 0.25 wt%, respectively, and an increase in wear area of up to 19% for 0.5 wt% MWCNTs. This could be explained by the fact that at a certain concentration of nanoadditive (0.5 wt%), clusters can form, which favor the agglomeration of nanotubes and can also act as abrasive particles, increasing wear [63]. Lijesh et al. [64] reported that MWCNTs dispersed without surfactant in mineral oil increased wear compared to mineral oil due to the agglomeration of MWCNTs. The optimum anti-friction and antiwear concentration for the h-BN nanoadditives is found to be the lowest (0.05 wt%). These results agree with those obtained for the contact angle (Figure 7b).

**Figure 9:**
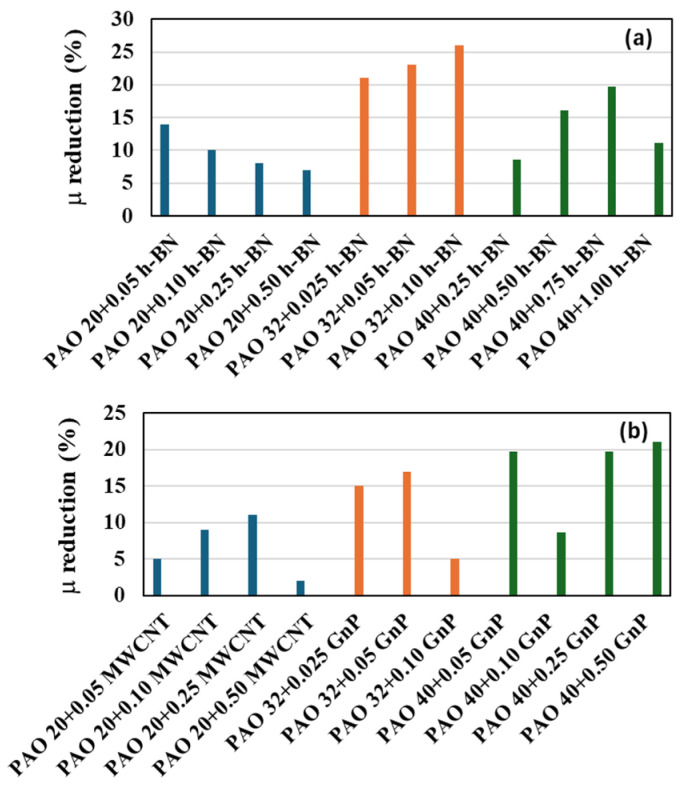
(**a**,**b**) Comparison among the reductions of the friction coefficient at different concentrations: PAO 20 + MWCNT (this work), PAO 20 + h-BN (this work), PAO 32 + h-BN [38], PAO 40 + h-BN [34], PAO 32 + GnP [38], PAO 40 + GnP [34].

In Figure 9, we have compared the friction coefficient reduction obtained in this work for PAO 20 + h-BN and for PAO 20 + MWCNT with previously published results [34,38] obtained, with the same apparatus and at the same experimental conditions, for PAO 32 + h-BN, PAO 32 + GnP, PAO40 + h-BN, and PAO40 + GnP. In Figure 9a, we can observe that for all the PAO base oils the friction coefficient is reduced due to the presence of h-BN, although the optimal composition (maximum reduction) is not the same: 0.05 wt% for PAO 20, 0.10 wt% for PAO 32 and 0.75 wt% for PAO 40. At a fixed composition of 0.05 wt% of h-BN, the reduction obtained is higher for PAO 32 than for PAO 20, and 0.50 wt% higher for PAO 40 than for PAO 20. It seems that the presence of h-BN is more effective when it is combined with PAO 32. Figure 9b shows the comparisons for PAO 20 + MWCNT with previous results [34,38] for PAO 32 + GnP and PAO 40 + GnP. For PAO 32 and PAO 40, a high reduction in the friction coefficient is obtained when a small concentration of GnP (0.05 wt%) is used. The reduction for this concentration is lower in the case of PAO 20 combined with the MWCNT nanoadditives. It can be concluded that the use of GnP can be more promising than MWCNT to reduce the friction coefficient when the base oil is a polyalphaolefin because we obtain a bigger friction coefficient reduction and lower viscosity increase [39].

**Figure 10:**
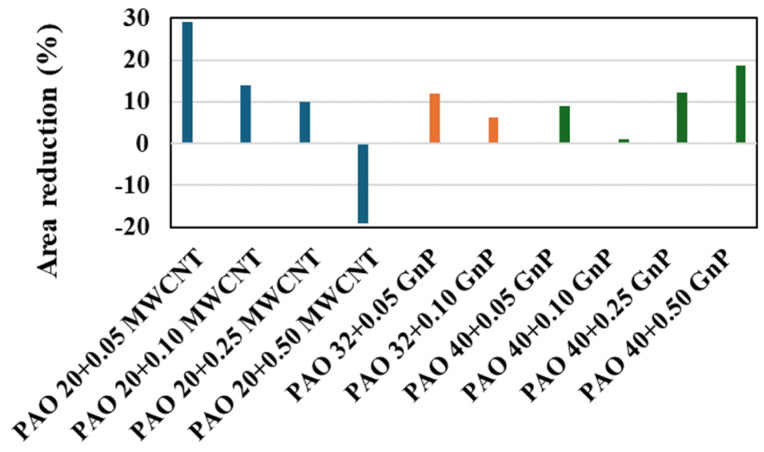
Comparison among the reductions of the transversal area of the wear track at different concentrations: PAO 20 + MWCNT nanolubricants (this work), PAO 32 + GnP [38], and PAO 40 + GnP [34].

Nevertheless, when this comparison at low nanoadditive concentrations is extended to the antiwear behavior, a small quantity of MWCNT (0.05 wt%) leads to the highest wear reductions in terms of the transversal area of the wear scar, as can be seen in Figure 10. Finally, we have compared the evolution with time of the refractive index reported in this work (Figure 5) with those previously published by Liñeira del Río et al. [34], concluding that in the case of the nanodispersion PAO 20 + MWCNT this property hardly varies compared to the trend of the PAO 40 + GnP nanodispersion. This fact seems to indicate that MWCNT is more stable than GnP when it is dispersed in PAO oils.

### 3.5. Anti-Friction and Antiwear Mechanisms

The average surface roughness (Ra) of worn surfaces of discs has been also analyzed to characterize the antiwear capability of the nanolubricants. Table 2 shows that surfaces lubricated with the lowest nanoadditive concentration (0.05 wt%) are smoother than those lubricated with PAO 20 base oil. Thus, the worn surface lubricated with PAO 20 base oil presented a Ra value of 22 nm, whereas in the track corresponding to the 0.05 wt% h-BN and MWCNT nanolubricants, the Ra was 12 and 15 nm, respectively, which leads to a 45% and 32% reduction in roughness, respectively. As a result, it can be concluded that a mending effect occurs due to the nanoadditive action at the lowest nanoadditive concentration. In the case of the h-BN, due to their lamellar structure, this effect is stronger than for the tubular shape of MWCNTs. While larger 2D sheets may not possess the capability to penetrate nanoscale valleys, they can still potentially contribute to smoothing out surface roughness by spanning across broader areas [65]. As the concentration of nanoadditives increases, the mending effect disappears, and the roughness in the wear track increases for both nanoadditives with respect to that caused by PAO 20 neat base oil. Tang et al. [66] reported that mildly aggregated structures, formed at low concentrations, efficiently accumulated within nanoscale valleys, leading to reduced wear on an amorphous carbon surface. Conversely, micron-sized aggregates, formed at higher additive concentrations, proved too large to penetrate voids, resulting instead in excessive plowing wear on the sliding surfaces of the contact.

**Figure 11:**
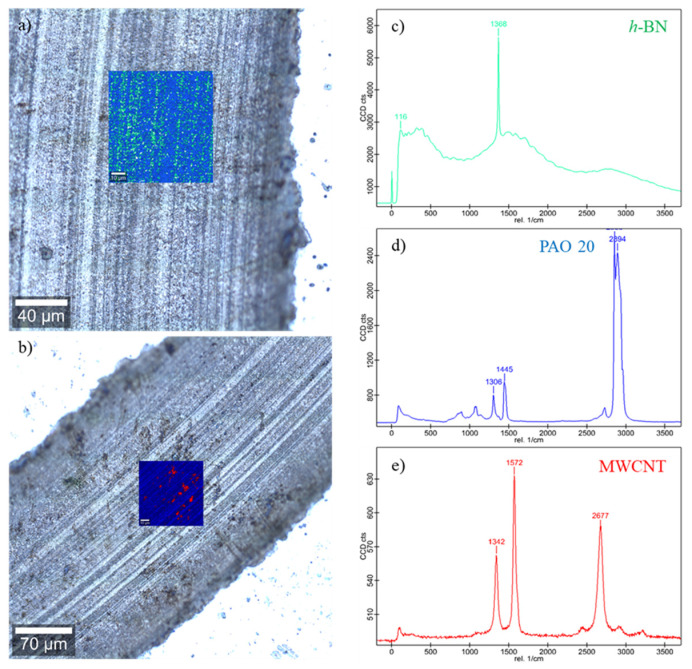
Micrograph and Raman mapping corresponding to the worn surface obtained with (**a**) 0.05 wt% h-BN and (**b**) 0.05 wt% MWCNT nanolubricants; (**c**) spectrum of the green area; (**d**) spectrum of the blue area; and (**e**) spectrum of the red area.

Figure 11 shows the Raman mapping and Raman spectra obtained on the worn surface tested with the optimum antiwear nanolubricant concentration for both nanoadditives: 0.05 wt% h-BN and 0.05 wt% MWCNTs. As can be observed, a high-energy active Raman band at 1368 cm^−1^ corresponding to h-BN nanoparticles [8,48] is detected along several furrows on the worn surface, indicating that both the mending effect and the formation of protective tribofilms containing h-BN (green areas in Figure 11a) take place. The tribofilms decrease the shearing resistance, favoring the sliding of both contacting surfaces. Similar results have been previously found by Charoo et al. [67] with an engine oil of grade SAE 20W-50 oil modified with h-BN nanoparticles. However, Raman mapping of the wear track lubricated with the 0.05 wt% MWCNT nanolubricant shows the nanotubes allowing inside the surface defects (red dots in Figure 11b). Thus, the MWCNTs at low concentrations reveal a mending effect that agrees with the surface roughness analysis.

**Figure 12:**
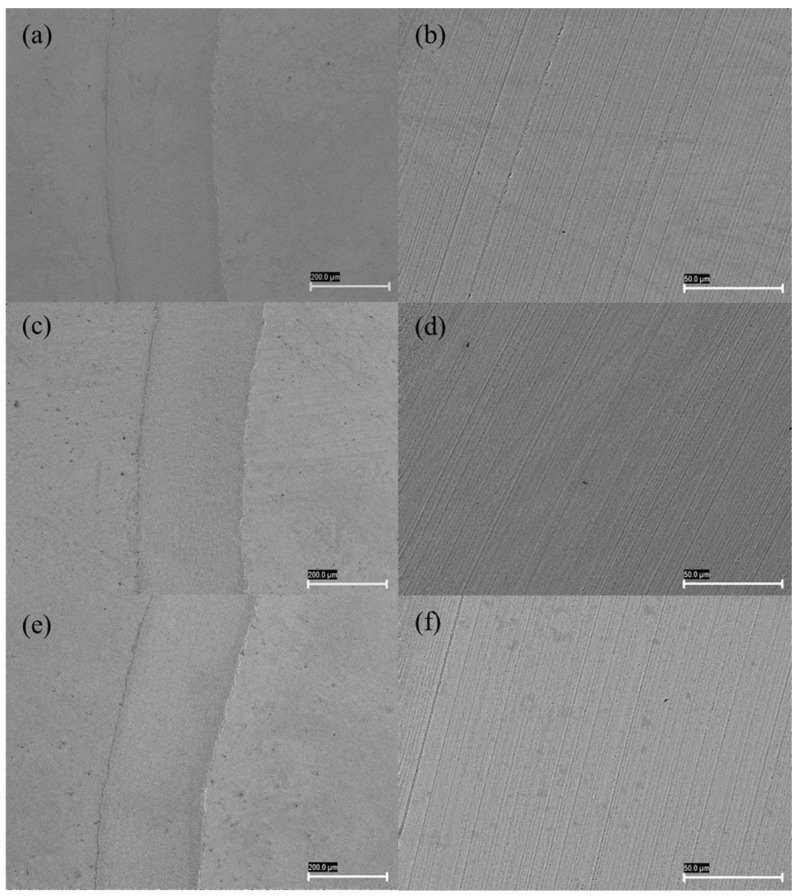
SEM images of the wear scars on the disc lubricated with PAO 20 base oil (**a**,**b**), 0.05 wt% h-BN (**c**,**d**) and 0.05 wt% MWCNTs nanolubricants (**e**,**f**) at different magnifications.

Figure 12 shows scanning electron microscopy (SEM) images of rubbing surfaces formed on steel discs lubricated with PAO 20 base oil and optimum antiwear concentrations of h-BN and MWCNTs taken at different magnifications. Low-magnification SEM images of all samples display smooth polished wear surfaces. A higher-magnification image details the nature of the wear mechanism taking place. Figure 12b,d,f shows that the worn surface of the steel disc has been lubricated with PAO 20 base oil and oil containing 0.05 wt% h-BN and 0.05 wt% MWCNTs, respectively. From this figure, it can be seen that the disc lubricated with base oil was very rough and showed deeper furrows and grooves. The higher magnification image (Figure 12f) shows the deposition of the MWCNTs in the plowing grooves. Dark patches observed on the abrasive grooves of the magnified wear surface reveal the mending effect produced by the MWCNTs. This explains the lower friction coefficient and wear rate values for nanolubricant containing 0.05 wt% MWCNT as an additive.

## 4. Conclusions

The incorporation of h-BN and MWCNTs nanoadditives exerted discernible effects on the thermophysical properties of the nanolubricants, notably increasing viscosity with the concentration of nanoadditives, up to a 47% increase over the PAO 20 base oil viscosity for the 0.5 wt% MWCNTs nanolubricant. This suggests the potential for tailored manipulation of lubricant characteristics through nanoadditive integration. Secondly, the tribological analysis revealed substantial insights into the efficacy of nanoadditives in improving the performance of the base oil. The friction-reducing performance and anti-wear properties of the PAO 20 base oil were significantly improved with the addition of MWCNTs and h-BN additives. The overall lubrication efficiency achieved by the 0.05 wt% h-BN nanolubricant led to a reduction in friction and wear of 14% and 37%, respectively. These reductions are lower for MWCNTs (5% and 29%, respectively); nevertheless, the dispersions that contain MWCNTs present better temporal stability. Furthermore, the comparative assessment of nanolubricants with distinct nanoadditives sheds light on the differential effects of MWCNTs and h-BN on lubricant performance, elucidating nuances in their tribological behavior. The analysis of wear surfaces suggested an antiwear mechanism of tribofilm formation combined with a mending effect for h-BN nanoparticles, while a pure mending effect was observed for MWCNTs at low concentrations.

## Figures and Tables

**Table 1:** Material properties of the tribological specimens.

	Steel Discs	Steel Balls
Material	AISI 52100	AISI 52100
Radius/mm	5	3
Ra/μm	0.02	0.05
Hardness	H_v30_ 190–210	58–66 Rockwell Scale

**Table 2:** Mean roughness surface (Ra) and its standard deviation measured on the wear scar of the steel disc after tests with all the lubricant samples.

PAO 20		0.05 wt%		0.1 wt%		0.25 wt%		0.5 wt%	
h-BN									
Ra/nm	σ/nm	Ra/nm	σ/nm	Ra/nm	σ/nm	Ra/nm	σ/nm	Ra/nm	σ/nm
		12	2	23	5	33	3	103	8
MWCNTs									
22	2	Ra/nm	σ/nm	Ra/nm	σ/nm	Ra/nm	σ/nm	Ra/nm	σ/nm
		15	5	27	12	47	8	88	9

## Data Availability

The original contributions presented in the study are included in the article/Supplementary Material, further inquiries can be directed to the corresponding author/s.

## Supplementary Materials

The following supporting information can be downloaded at: https://www.mdpi.com/article/10.3390/nano14131101/s1, Table S1: Experimental densities, *ρ*^a^, determined with the Stabinger SVM 3000 densimeter for the base oil (PAO 20) and the nanolubricants at several temperatures, T^b^, and 0.0991 MPa^c^.; Table S2: Experimental viscosity, η^a^, determined with Stabinger rotational viscometer for the base oil (PAO 20) and for the nanolubricants at 0.0991 MPa^b^ at different temperatures T^c^.; Table S3: Parameters obtained (A, B and C) for Equation (1) for PAO 20 base oil and each nanolubricant and average absolute deviation (AAD%) between experimental and correlated viscosity values. Figure S1. Raman spectra of nanolubricants with 0.5 wt% h-BN and 0.5 wt% MWCNT.
