# Supplementary material for: Performance and Antiwear Mechanism of 1D and 2D Nanoparticles as Additives in a Polyalphaolefin
Source: Nanomaterials (Basel). 2024 Jun 27;14(13):1101. doi: 10.3390/nano14131101 (PMC11243078; doi:10.3390/nano14131101)
Supplement: Supplementary file 1 [file nanomaterials-14-01101-s001.zip › nanomaterials-3040059-supplementary.pdf]

nanomaterials

Supplementary Material

Performance and Antiwear Mechanism of 1D and 2D Nanoparticles as Additives in a Polyalphaolefin

María J. G. Guimarey [1,*], Antía Villamayor [2], Enriqueta R. López [1] and María J. P. Comuñas [1]

[1] Laboratory of Thermophysical and Tribological Properties, NaFoMat Group, Department of Applied Physics, Faculty of Physics, University of Santiago de Compostela, 15782, Santiago de Compostela, Spain; enriqueta.lopez@usc.es (E.R.L.); mariajp.comunas@usc.es (M.J.P.C.)
[2] Physics of Surfaces and Materials Unit, Tekniker, Basque Research and Technology Alliance (BRTA), C/Iñaki Goenaga 5, 20600 Eibar, Spain; antia.villamayor@tekniker.es
* Correspondence: mariajesus.guimarey@usc.es; Tel.: +34-881814114

Table S1. Experimental densities, ρ^a, determined with the Stabinger SVM 3000 densimeter for the base oil (PAO 20) and for the nanolubricants at several temperatures, T^b, and 0.0991 MPa[c].

T/K	ρ/g cm^{-3}	*T*/K	ρ/g cm^{-3}	*T*/K	ρ/g cm^{-3}
		PAO 20 base oil			
278.15	0.8471	313.15	0.8261	348.15	0.8051
283.15	0.8441	318.15	0.8232	353.15	0.8021
288.15	0.8410	323.15	0.8202	358.15	0.7990
293.15	0.8381	328.15	0.8172	363.15	0.7960
298.15	0.8351	333.15	0.8141	368.15	0.7930
303.15	0.8321	338.15	0.8111	373.15	0.7900
308.15	0.8291	343.15	0.8081		
		99.95 wt% PAO 20 + 0.05 wt% MWCNT			
278.15	0.8471	313.15	0.8262	348.15	0.8053
283.15	0.8441	318.15	0.8232	353.15	0.8023
288.15	0.8410	323.15	0.8203	358.15	0.7993
293.15	0.8380	328.15	0.8173	363.15	0.7962
298.15	0.8351	333.15	0.8143	368.15	0.7932
303.15	0.8321	338.15	0.8113	373.15	0.7902
308.15	0.8291	343.15	0.8083		
		99.9 wt% PAO 20 + 0.1 wt% MWCNT			
278.15	0.8473	313.15	0.8264	348.15	0.8055
283.15	0.8443	318.15	0.8234	353.15	0.8025
288.15	0.8412	323.15	0.8205	358.15	0.7995
293.15	0.8382	328.15	0.8175	363.15	0.7965
298.15	0.8353	333.15	0.8145	368.15	0.7935
303.15	0.8323	338.15	0.8115	373.15	0.7905
308.15	0.8293	343.15	0.8085		
		99.75 wt% PAO 20 + 0.25 wt% MWCNT			
278.15	0.8480	313.15	0.8270	348.15	0.8062
283.15	0.8450	318.15	0.8241	353.15	0.8031

288.15	0.8420	323.15	0.8211	358.15	0.8001
293.15	0.8390	328.15	0.8181	363.15	0.7971
298.15	0.8360	333.15	0.8152	368.15	0.7941
303.15	0.8330	338.15	0.8122	373.15	0.7911
308.15	0.8300	343.15	0.8092		
99.5 wt% PAO 20 + 0.5 wt% MWCNT					
278.15	0.8490	313.15	0.8280	348.15	0.8072
283.15	0.8461	318.15	0.8251	353.15	0.8042
288.15	0.8431	323.15	0.8221	358.15	0.8012
293.15	0.8400	328.15	0.8191	363.15	0.7982
298.15	0.8370	333.15	0.8162	368.15	0.7952
303.15	0.8340	338.15	0.8132	373.15	0.7922
308.15	0.8310	343.15	0.8102		
99.95 wt% PAO 20 + 0.05 wt% h-BN					
278.15	0.8471	313.15	0.8262	348.15	0.8053
283.15	0.8441	318.15	0.8233	353.15	0.8023
288.15	0.8411	323.15	0.8203	358.15	0.7993
293.15	0.8381	328.15	0.8174	363.15	0.7962
298.15	0.8351	333.15	0.8144	368.15	0.7932
303.15	0.8322	338.15	0.8114	373.15	0.7902
308.15	0.8292	343.15	0.8084		
99.9 wt% PAO 20 + 0.1 wt% h-BN					
278.15	0.8477	313.15	0.8268	348.15	0.8059
283.15	0.8447	318.15	0.8239	353.15	0.8028
288.15	0.8417	323.15	0.8209	358.15	0.7998
293.15	0.8387	328.15	0.8179	363.15	0.7968
298.15	0.8357	333.15	0.8149	368.15	0.7938
303.15	0.8327	338.15	0.8119	373.15	0.7908
308.15	0.8298	343.15	0.8089		
99.75 wt% PAO 20 + 0.25 wt% h-BN					
278.15	0.8484	313.15	0.8275	348.15	0.8064
283.15	0.8454	318.15	0.8245	353.15	0.8034
288.15	0.8424	323.15	0.8215	358.15	0.8004
293.15	0.8394	328.15	0.8185	363.15	0.7974
298.15	0.8364	333.15	0.8155	368.15	0.7943
303.15	0.8335	338.15	0.8125	373.15	0.7913
308.15	0.8305	343.15	0.8095		
99.5 wt% PAO 20 + 0.5 wt% h-BN					
278.15	0.8498	313.15	0.8288	348.15	0.8077
283.15	0.8468	318.15	0.8258	353.15	0.8047
288.15	0.8437	323.15	0.8228	358.15	0.8017
293.15	0.8407	328.15	0.8198	363.15	0.7986
298.15	0.8377	333.15	0.8168	368.15	0.7956
303.15	0.8348	338.15	0.8138	373.15	0.7926
308.15	0.8318	343.15	0.8108		

[a] Combined expanded density uncertainty is U_c (ρ)= $5{\cdot}10^{-4}$ g cm^{-3}; [b] expanded temperature uncertainty is U(T) = 0.02 K in the range from 288.15 to 378.15 K and outside this range 0.05 K and [c] expanded pressure uncertainty is U (p) = 0.0005 MPa (0.95 level of confidence).

Table S2. Experimental viscosity, η^a, determined with Stabinger rotational viscometer for the base oil (PAO 20) and for the nanolubricants at 0.0991 MPa[b] at different temperatures T^c.

T^c/K	η/mPa s	T/K	η/mPa s	T/K	η/mPa s
		PAO 20 base oil			
278.15	957	313.15	121	348.15	31.0
283.15	670	318.15	96.6	353.15	26.5
288.15	482	323.15	77.8	358.15	22.8
293.15	353	328.15	63.4	363.15	19.8
298.15	264	333.15	52.2	368.15	17.3
303.15	200	338.15	43.5	373.15	15.2
308.15	155	343.15	36.5		
		99.95 wt% PAO 20 + 0.05 wt% MWCNT			
278.15	981	313.15	127	348.15	32.2
283.15	688	318.15	101	353.15	27.5
288.15	495	323.15	81.5	358.15	23.6
293.15	364	328.15	68.8	363.15	20.4
298.15	273	333.15	54.6	368.15	17.8
303.15	208	338.15	45.4	373.15	15.6
308.15	162	343.15	38.1		
		99.9 wt% PAO 20 + 0.1 wt% MWCNT			
278.15	1010	313.15	133	348.15	33.3
283.15	709	318.15	106	353.15	28.3
288.15	513	323.15	84.9	358.15	24.3
293.15	379	328.15	69.0	363.15	21.0
298.15	286	333.15	56.7	368.15	18.2
303.15	219	338.15	47.1	373.15	15.9
308.15	169	343.15	39.4		
		99.75 wt% PAO 20 + 0.25 wt% MWCNT			
278.15	1098	313.15	151	348.15	36.8
283.15	774	318.15	119	353.15	31.1
288.15	566	323.15	95.8	358.15	26.6
293.15	425	328.15	77.6	363.15	22.8
298.15	324	333.15	63.5	368.15	19.7
303.15	249	338.15	52.4	373.15	17.1
308.15	192	343.15	43.7		
		99.5 wt% PAO 20 + 0.5 wt% MWCNT			
278.15	1314	313.15	193	348.15	43.9
283.15	948	318.15	151	353.15	36.8
288.15	714	323.15	120	358.15	31.3
293.15	550	328.15	96.4	363.15	26.9
298.15	424	333.15	77.9	368.15	23.3
303.15	323	338.15	63.8	373.15	20.2
308.15	248	343.15	52.5		
		99.95 wt% PAO 20 + 0.05 wt% h-BN			
278.15	962	313.15	122	348.15	31.1
283.15	674	318.15	96.8	353.15	26.6
288.15	484	323.15	77.9	358.15	22.9
293.15	354	328.15	63.5	363.15	19.9
298.15	265	333.15	52.4	368.15	17.4
303.15	201	338.15	43.6	373.15	15.3
308.15	155	343.15	36.7		

		99.9 wt% PAO 20 + 0.1 wt% h-BN			
278.15	959	313.15	122	348.15	31.1
283.15	673	318.15	96.9	353.15	26.6
288.15	483	323.15	78.0	358.15	23.0
293.15	354	328.15	63.6	363.15	20.0
298.15	264	333.15	52.4	368.15	17.4
303.15	201	338.15	43.7	373.15	15.3
308.15	155	343.15	36.7		
		99.75 wt% PAO 20 + 0.25 wt% h-BN			
278.15	959	313.15	122	348.15	31.3
283.15	674	318.15	97.3	353.15	26.8
288.15	484	323.15	78.4	358.15	23.1
293.15	355	328.15	63.9	363.15	20.0
298.15	266	333.15	52.6	368.15	17.5
303.15	202	338.15	43.8	373.15	15.4
308.15	156	343.15	36.9		
		99.5 wt% PAO 20 + 0.5 wt% h-BN			
278.15	966	313.15	123	348.15	31.6
283.15	678	318.15	98.0	353.15	27.1
288.15	487	323.15	79.0	358.15	23.4
293.15	357	328.15	64.4	363.15	20.3
298.15	267	333.15	53.1	368.15	17.8
303.15	203	338.15	44.3	373.15	15.7
308.15	157	343.15	37.2		

[a] Combined relative expanded viscosity uncertainty is U_c (η) = 1%; [b] expanded pressure uncertainty is U (p) = 0.0005 MPa and [c] expanded temperature uncertainty is U(T) = 0.02 K in the range from 288.15 to 378.15 K and outside this range 0.05 K.

Table S3. Parameters obtained (*A*, *B* and *C*) for Eq. (1) for PAO 20 base oil and each nanolubricant and average absolute deviation (*AAD*%) between experimental and correlated viscosity values.

Sample	*A* / mPa s^{-1}	*B* / K	*C* / K	*AAD*%
PAO 20 base oil	0.0423	1350.2	143.55	0.06
99.95 wt% PAO 20 + 0.05 wt% MWCNT	0.0433	1342.8	144.07	0.06
99.9 wt% PAO 20 + 0.1 wt% MWCNT	0.0436	1342.0	144.00	0.06
99.75 wt% PAO 20 + 0.25 wt% MWCNT	0.0433	1346.8	143.62	0.08
99.5 wt% PAO 20 + 0.5 wt% MWCNT	0.0474	1321.2	145.05	0.08
99.95 wt% PAO 20+ 0.05 wt% h-BN	0.0301	1488.6	134.83	0.10
99.9 wt% PAO 20 + 0.1 wt% h-BN	0.0239	1584.6	129.35	0.06
99.75 wt% PAO 20 + 0.25 wt% h-BN	0.0104	1954.0	109.13	0.11
99.5 wt% PAO 20+ 0.5 wt% h-BN	0.0022	2731.6	73.358	0.40

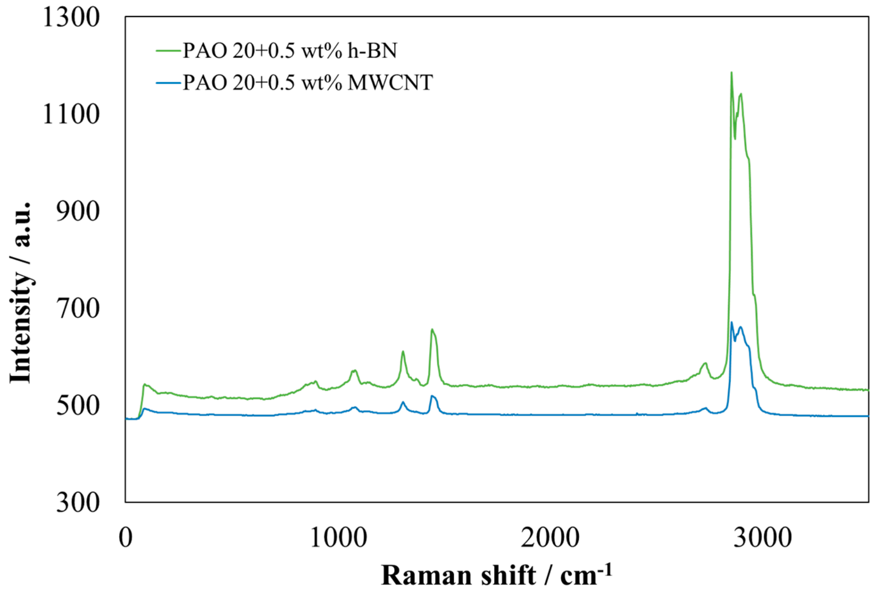

Figure S1. Raman spectra of nanolubricants with 0.5 wt% h-BN and 0.5 wt% MWCNT.
